# Social Networking Site Use During the COVID-19 Pandemic and Its Associations With Social and Emotional Well-being in College Students: Survey Study

**DOI:** 10.2196/26513

**Published:** 2021-09-07

**Authors:** Alison B Tuck, Renee J Thompson

**Affiliations:** 1 Department of Psychological and Brain Sciences Washington University in St Louis St Louis, MO United States

**Keywords:** social media, social networking sites, COVID-19, loneliness, well-being

## Abstract

**Background:**

Social distancing during the COVID-19 pandemic has reduced the frequency of in-person social interactions. College students were highly impacted, since many universities transferred curriculum from in-person to entirely online formats, physically separating students with little notice. With social distancing, their use of social networking sites (SNSs) likely changed during the COVID-19 pandemic, possibly holding implications for well-being.

**Objective:**

This study aimed to determine (1) how components of SNS use (ie, weekly frequency, time per day, habitual use, engagement, enjoyment, addiction, and emotional impact) changed from before to during COVID-19, (2) how these changes in SNS use were associated with pandemic-related social and emotional well-being, and (3) how SNS use and changes in use during the pandemic were associated with loneliness.

**Methods:**

College students (N=176) were surveyed during the time when their university campus in the United States was operating online. Participants completed the same SNS use questionnaires twice, once with regard to the month preceding the onset of COVID-19 and again with regard to the month since this time. They also reported the extent to which they experienced perceived change in social support resulting from the pandemic, pandemic-related stress, and general loneliness.

**Results:**

After the onset of COVID-19, participants showed an increase in daily time spent on SNSs (*t_169_*=5.53, *d*=0.42, *P*<.001), habitual use (*t_173_*=3.60, *d*=0.27, *P*<.001), and addiction (*t_173_*=4.96, *d*=0.38, *P*<.001); further, enjoyment on SNSs decreased (*t_173_*=–2.10, *d*=–0.16, *P*=.04) and the emotional impact of SNS activities became more negative (*t_172_*=–3.76, *d*=–0.29, *P*<.001). Increased perceived social support during COVID-19 was associated with changes in frequency of SNS use, time per day, addiction, and engagement (*r*>0.18 for all). Pandemic-related stress was associated with changes in SNS addiction and the extent to which one’s SNS content was related to the pandemic (*r*>0.20 for all). Loneliness was positively associated with SNS addiction (*r*=0.26) and negatively associated with SNS engagement (*r*=–0.19) during the pandemic. Loneliness was also negatively associated with changes in habit and engagement (*r*<–0.15 for all).

**Conclusions:**

Findings suggest that components of SNS use are associated with both positive and negative pandemic-related social outcomes, but largely negative pandemic-related emotional outcomes. Further, some components of SNS use are positively associated with loneliness (eg, addiction) while others show a negative association (eg, engagement). These findings provide a more nuanced picture of how SNS use is associated with social and emotional well-being during the time of a global health crisis when in-person interactions are scarce.

## Introduction

The infectious respiratory disease COVID-19 was first recognized in Wuhan, China, in December of 2019 [1]. On March 11, 2020, the World Health Organization declared COVID-19 a pandemic. As of February 17, 2021, COVID-19 has infected over 109 million individuals across the globe, and over 2.4 million deaths caused by COVID-19 have been reported to date [2]. Approximately a quarter of these cases are from the United States, representing the largest number of cases compared to any other country across the globe [2].

Like many widespread outbreaks of infectious diseases [3], the COVID-19 pandemic has been associated with negative psychological outcomes, such as increased rates of depression, anxiety, and stress in the general population [4]. Several researchers theorize that these mental health outcomes are a result of social distancing—the act of physically isolating and not interacting in person with others to reduce the risk of spreading the disease [5,6]. A possible explanation of this link is that less frequent in-person social interactions are associated with lower psychological well-being [7]. With social distancing being recommended and sometimes enforced in communities across the globe, it is important to examine how individuals are coping with, and may be compensating for, their less frequent in-person interactions. In a technology-driven world, social networking sites (SNSs) may be the best alternative for many people. The central goals of this investigation are to examine how SNS use has changed during the COVID-19 pandemic and to analyze how these changes in SNS use are related to pandemic-related social and emotional well-being as well as loneliness.

SNSs refer to a specific type of social media in which “communities” are formed consisting of public or semipublic profiles and where individuals can regulate with whom they connect as well as browse the connections of others [8]. Use of SNSs has been assessed with a variety of measures, most of which almost exclusively assess time per day spent on SNSs. Studies using these measures have yielded mixed results [9]. Some studies show positive associations between time spent on SNSs and negative mental health outcomes (ie, depression and anxiety) [9,10]. Other studies show no such associations. For example, a recent 8-year longitudinal study found no association between daily time spent on SNSs and depression or anxiety [11], stressing the need for researchers to evaluate use of SNSs beyond a focus on screen time.

In addition to examining time spent on SNSs, Turel and Serenko [12] developed a model for understanding a wide range of components of SNS use and how they lead to either favorable or adverse outcomes: SNS addiction, engagement with SNSs, time per day, enjoyment on SNSs, and habitual SNS use. According to their model, SNS addiction*—*defined as a dependency on SNSs that results in an obsessive pattern of SNS seeking and use that interferes with engagement in other important activities—is an adverse and pathological outcome [12]. Conversely, engagement with SNSs*—*defined as individuals caring about SNSs and making them a significant aspect of their lives that they can control—is seen as a favorable, nonpathological outcome [12].

Turel and Serenko theorize that enjoyment on SNSs, or an individual’s intrinsic motivation for using SNSs simply because of their emotional rewards, is what leads to high SNS engagement [12]. However, enjoyment on SNSs seems to be a double-edged sword, as this variable, along with time per day spent on SNSs, is theorized to lead to habitual SNS use. Turel and Serenko hypothesize that habitual use occurs when individuals use SNSs automatically in certain contexts due to some learned association. This habitual use can often lead to SNS addiction, the pathological outcome. Consistent with their theorizing, SNS addiction has been found to be positively associated with decreased psychological well-being (eg, depressive symptomology) [13]. In this regard, Turel and Serenko’s model provides a detailed overview of the use and consequences of SNSs. However, researchers have not analyzed how these various SNS use components may have changed as a result of social distancing caused by widespread disease. With current social distancing initiatives making it so that SNS use may be one of the most common forms of social interaction, individuals might be using SNSs more during the pandemic while possibly not reaping the same emotional benefits from its use.

An important next step for SNS research is to examine how SNS use is associated with emotional experiences. Analyzing the emotions experienced by individuals on SNSs expands the literature by clarifying when in-the-moment SNS use might be positive and when it might be negative. This moves the field beyond measuring associations between SNS use and depression and anxiety—symptoms of disorders that have relatively low base rates—and allows us to analyze the short-term emotional influence of SNSs and how SNSs affect quality of life. For example, perhaps there are periods of time in which individuals experience more positive emotions while on SNSs, and other times in which they experience more negative emotions. These short-term emotional impacts, when experienced regularly, could have important implications for psychological well-being.

Emerging literature demonstrates that SNS use is associated with negative emotional experiences during the current pandemic. For instance, weekly frequency of exposure to COVID-19–related content on SNSs was associated with higher levels of general psychological distress in a large Chinese sample [14]. Some researchers have postulated that the misinformation being spread across SNSs about the pandemic (eg, conspiracy theories) leads to increases in stress, anxiety, and panic in relation to COVID-19 [14-16]. Evidence and theory that exposure to COVID-19–related SNS content correlates with negative mental health outcomes suggests that SNS use during COVID-19 might be associated with decreases in social and emotional well-being during the pandemic.

Another possible negative consequence of the pandemic is increased rates of loneliness. Indeed, a recent large-scale study of adults found that approximately 36% of participants endorsed sometimes or often feeling lonely during the pandemic [17]. Loneliness is conceptualized as a wish to feel closer to others when individuals otherwise feel isolated [18]. This sense of isolation may be the result of being physically separate from others—as could particularly be the case during social distancing initiatives during COVID-19—or of feeling emotionally isolated. Of note, compared to midlife and older adults, younger adults are particularly vulnerable to experiencing loneliness when they have a diminished *quantity* (versus *quality*) of social engagements [19]. Given that many college students experienced their universities abruptly cease in-person operations during COVID-19, this group experienced great decreases in social interactions and likely experienced increases in loneliness.

Ample research suggests that loneliness is associated with SNS use. For example, those who have few in-person social interactions and who use SNSs a great amount report higher levels of loneliness than other groups of individuals, including those who have few in-person social interactions and who use SNSs only a small amount [20]. Research testing causal models suggest that loneliness is the cause of increased SNS use and not that SNS use is the cause of loneliness [21]. Taken together, this literature suggests that loneliness may be associated with particularly high increases in college students’ SNS use during the pandemic, when in-person social interactions are scarce.

This investigation had three primary aims. The first aim was to determine how SNS use changed, overall, from before to after the onset of the COVID-19 pandemic. Consistent with Turel and Serenko’s model [12], we hypothesized that components of SNS use associated with addiction—which is associated with worse psychological well-being—would increase from pre– to during COVID-19. Specifically, compared to pre–COVID-19, we hypothesized that time per day spent on SNSs, habit, and addiction increased during COVID-19. In addition, we expected that SNS engagement decreased, and we did not expect enjoyment to significantly change during this period. Consistent with prior research and theory, we also expected that SNS frequency increased and that the average emotional impact from SNS use decreased during COVID-19.

The second aim was to examine how changes in SNS use were associated with pandemic-related social well-being (ie, changes in perceived social support during the pandemic) and pandemic-related emotional well-being (ie, pandemic-related stress). Again, consistent with Turel and Serenko’s model and other research, we expected that poor pandemic-related social and emotional well-being would be positively associated with changes in SNS frequency, time per day, habit, addiction, and the percentage that one’s SNS content was related to the pandemic. Further, we hypothesized that lower levels of pandemic-related social and emotional well-being would be associated with decreases in SNS engagement and average emotional impact from SNSs. Importantly, we expected each of the associations between SNS use and pandemic-related emotional well-being to remain significant after controlling for general distress, which was indexed by depressive symptoms.

To expand upon the extant literature regarding associations between SNS use, COVID-19, and loneliness, the third aim was to examine the associations between loneliness and both (1) components of SNS use during COVID-19 and (2) changes in components of SNS use from pre– to during COVID-19. In this investigation, loneliness was conceptualized as an outcome that was not specifically related to the pandemic. In other words, although we expect that loneliness increased during the pandemic, loneliness was examined as a general measure. Given that loneliness is also a type of psychological distress indicative of poor psychological well-being, consistent with Turel and Serenko’s model, we hypothesized that loneliness would be positively associated with changes in time spent on SNSs, frequency of SNS use, habitual SNS use, and SNS addiction and that it would be negatively associated with engagement with SNSs, enjoyment on SNSs, and the average emotional impact of SNSs. We expected parallel associations of SNS use during COVID-19 specifically (eg, loneliness would be positively associated with time spent on SNSs during COVID-19). Importantly, we expected that all of the relationships between loneliness and SNS use components would hold even after accounting for social anxiety, which is positively associated with loneliness [22].

This study focused on SNS use among college students. Approximately 90% of young adults in the United States aged 18 to 29 years use SNSs, representing the largest adult group to engage with these platforms [23]. Further, college students shared unique experiences early during the COVID-19 outbreak in that colleges closed midterm across the United States. As a result, college students were specifically and greatly impacted by disturbances to their normal social functioning during the pandemic, possibly above and beyond any other adult group. We capitalized on this clearly defined disruption among this group (ie, before universities closed versus during university closures) to examine the impact of COVID-19 on SNS use. Finally, college students are at an increased risk for various psychological disorders, including depression, anxiety, and substance use disorders, at rates higher than their older peers [24,25]. In addition, significantly more college-aged adults endorse serious psychological distress, such as feeling nervous or hopeless, compared to adults aged 22 to 34 years [26]. This trend may, in part, be explained by college students facing many unique stressors, such as academic pressure and first-time separation from family [26,27]. Of note, although college students are not at increased risk for developing a psychiatric disorder compared to their peers who do not attend college, they are significantly less likely to receive mental health treatment [28,29]. The shortage of mental health treatment available to college students has been deemed to be a mental health crisis [30,31]. SNSs may provide a needed venue for college students to engage in self-disclosure and establish social connection when they cannot acquire formal mental health treatment [32]. For these reasons, we think examining college students during the COVID-19 pandemic will provide a more thorough understanding of the role of SNSs during this challenging time.

## Methods

### Recruitment

The entire study was administered online from April 14 to 24, 2020. Undergraduate students in psychology courses learned about the study via a university portal that lists active studies. The portal was open to all undergraduate students enrolled in psychology courses at the university, and it provided students with a hyperlink to access the study. The first webpage of the study presented interested individuals with an informed consent form. Those who consented were directed to complete a demographics questionnaire followed by the rest of the study measures. All participants completed the study within a time frame of about one hour and received one hour of course research credit for their participation. All study procedures were reviewed and approved by the Institutional Review Board at Washington University in St Louis, Missouri.

### Procedures

Cases of COVID-19 surged in the United States in March of 2020. Coincidentally, this initial surge began during the university’s spring break, which took place from March 8 to 21, 2020, when almost all students leave campus. During this time, students were told that the university would no longer be holding in-person instruction, and they were not allowed to return to campus. As such, all participants in this study shared the same unique experience of not just being students who use SNSs quite regularly, but also of COVID-19 having the largest impact on daily life *after* spring break with a clear date delineating “pre–COVID-19” and “during COVID-19” time frames.

In this one-part study, we administered four sets of measures. First, we administered the same series of SNS use self-report measures twice; the only difference was the period of time that students considered when completing them. For the first set of SNS use measures, students answered with regard to the month preceding their spring break (ie, “pre–COVID-19,” from February 7 to March 7, 2020, before receiving the news that instruction was transitioning online). For the second set of SNS use measures, they answered the questions with regard to the time since spring break (ie, “during COVID-19”), which ranged anywhere from 3 weeks and 1 day earlier to 4 weeks and 4 days earlier. In this second set of SNS use questions, participants were additionally asked to report on the extent to which the content on their SNSs was related to the pandemic. The third set of measures included three measures assessing pandemic-related social and emotional well-being. Finally, the fourth set of measures included three psychological distress measures, the order of which were randomly presented across participants.

### Measures

#### Components of Social Networking Site Use

##### Overview

We asked participants to report on various components of their SNS use: the frequency with which they visited specific SNSs (ie, weekly frequency), time per day, habit, engagement, enjoyment, and addiction. We also assessed the average emotional impact of discrete SNS activities (eg, looking at memes) as well as how much one’s SNS content to which they were exposed was related to COVID-19 after the outbreak. How we measured these SNS components is described in detail below.

##### Weekly Frequency

We assessed weekly frequency of SNS use by presenting participants with a list of seven SNSs: Facebook, Instagram, Twitter, Snapchat, Reddit, Tumblr, and LinkedIn. These sites were selected based on two selection criteria: sites on which the people in one’s network are people whom one is likely to know “in real life” and/or there is a significant focus on both consuming and commenting on content. Therefore, sites on which followers are unlikely to know one another in real life and on which there is not a significant focus on commenting on content (eg, TikTok) were not included. In addition, sites that are strictly text or communication based (eg, Facebook Messenger) were also not included. Participants endorsed how frequently they used each of the seven sites in a typical week of the given time frame (ie, weekly frequency). These items were scored on an experimenter-generated 8-point Likert scale from 1 (never) to 8 (7+ times per day). Values were summed across the seven SNSs, such that total weekly frequency scores could range from 7 to 56.

iPhones have a Screen Time function in phone settings that provides a breakdown of cell phone use activity, including average daily time spent on one’s phone and weekly total screen time; a comparable feature is not available on Android or other mobile cellular devices. Those with iPhones (156/176, 88.6%) reported these two values. Our weekly frequency variable was positively correlated with participants’ “weekly total screen time” on their iPhones (*r*=0.26, *P*=.002), suggesting that participants were able to accurately estimate how much they visit their phones (and SNSs) each week.

##### Time per Day

To determine how much time participants spent on each of the seven SNSs (ie, time per day), they were directly asked to report “how much time in a typical day” in the given time frame they had used each of the sites. For each participant, the total minutes endorsed for each of the seven sites were summed to compute a total time per day score. Our total time per day variable was significantly positively correlated with iPhone reports of “average daily time” (*r*=0.41, *P*<.001), suggesting that participants were able to accurately estimate how much time they spend on their phones each day.

##### Habit, Engagement, and Enjoyment

Habitual SNS use, engagement with SNSs, and enjoyment on SNSs were each assessed using the corresponding subscales developed by Turel and Serenko. For each subscale, we modified wording to refer to use across all “social networking platforms” rather than to address one specific site (eg, “Using social networking platforms has become automatic to me”). Participants were asked, “During [time frame], to what extent did you agree with the following statements?” Participants endorsed each item using a 5-point Likert scale from 1 (strongly disagree) to 5 (strongly agree). These three subscales have been validated on college student samples [12] and are described below.

Habitual SNS use (ie, habit) was assessed by three items: “Using social networking platforms has become automatic to me,” “Using social networking platforms is natural to me,” and “When I want to interact with friends and relatives, using social networking platforms is an obvious choice for me.” The three values were averaged to compute a habit score. Internal consistency scores for habit were good (pre–COVID-19: *α*=.76; during COVID-19: *α*=.85).

Engagement with SNSs (ie, engagement) was assessed by three reverse-coded items: “It would not matter to me if I never used social networking platforms again,” “The less I have to do with social networking platforms, the better,” and “Social networking platforms are unimportant in my life.” The three values were averaged to compute an engagement score. Internal consistency scores for engagement were good (pre–COVID-19: *α*=.80; during COVID-19: *α*=.85).

Enjoyment on SNSs (ie, enjoyment) was assessed by five items: “Using social networking platforms is enjoyable,” “Using social networking platforms is pleasurable,” “Using social networking platforms is fun,” “Using social networking platforms is exciting,” and “Using social networking platforms is interesting.” The five values were averaged to compute an enjoyment score. Internal consistency scores for engagement were good (pre–COVID-19: *α*=.86; during COVID-19: *α*=.89).

##### Addiction

SNS addiction (ie, addiction) was assessed using an adapted version of the Bergen Facebook Addiction Scale [33] that was originally developed by Shensa et al [34]. Using a 5-point Likert scale from 0 (very rarely) to 4 (very often), participants indicated the frequency with which they experienced the following six symptoms: “Spent a lot of time thinking about social networking platforms or planned use of social networking platforms,” “Felt an urge to use social networking platforms more and more,” “Used social networking platforms in order to forget about personal problems,” “Tried to cut down on your use of social networking platforms without success,” “Become restless or troubled if you have been prohibited from using social networking platforms,” and “Used social networking platforms so much that it had a negative impact on your job/studies.” Items were summed and could range from 0 to 24. This scale has been validated with a nationally representative young adult (aged 19-32 years) sample [34]. The items in this scale had good internal consistency for both administrations (pre–COVID-19: *α*=.89; during COVID-19: *α*=.89).

##### Average Emotional Impact From SNS Activities

To assess emotional outcomes specifically resulting from SNS use, participants were additionally presented with a 45-item list of discrete SNS activities (eg, “Read or watched news with content that I found negative or upsetting” and “Commented positively or supportively on other's post(s)”). These items were developed through informal undergraduate focus groups and experimenter-generated items. When applicable, parallel activities were developed for items such that each activity included a positive, negative, and neutral valence (eg, “Shared a post(s) about positive events or emotions,” “Shared a post(s) about negative events or emotions,” and “Shared a post(s) about neutral (neither positive nor negative) events or emotions”). The list was presented in a random order for each participant. For each activity, participants were first asked to indicate whether they had engaged in each activity during the given time frame. For all activities endorsed, participants were then asked to indicate “what impact each of these activities had on your emotions, on average” during the given time frame on a 7-point Likert scale from 1 (made me feel really bad) to 7 (made me feel really good). These scores were summed and divided by the total number of activities the participant endorsed to calculate an average emotional impact score for each person at each time frame. Of note, individuals’ average emotional impact was significantly positively associated with enjoyment on SNSs both pre–COVID-19 (*r*=0.35, *P*<.001) and during COVID-19 (*r*=0.29, *P*<.001), suggesting that our emotional impact variable was able to adequately capture the emotional influence of SNS activities. It is important to note that this variable can also be thought of as a form of emotional well-being, although it is largely utilized as a predictor variable in this study.

##### COVID-19 SNS Content

To assess COVID-19–related content, participants were administered one experimenter-generated question about the extent to which their SNS content was related to COVID-19. They were asked, “Since the end of spring break (March 23rd), what percentage of your SNS content would you estimate is COVID-19 related?” Participants reported what percentage they felt their SNS content was pandemic related in a text box.

#### Pandemic-Related Social and Emotional Well-being Measures

We administered two additional experimenter-generated measures to assess social and emotional well-being specific to the period of time during the COVID-19 pandemic: change in perceived social support and pandemic-related stress.

##### Change in Perceived Social Support

We operationalized pandemic-related social well-being as “change in perceived social support” since the onset of COVID-19. It was assessed with two items: “Prior to the COVID-19 outbreak, how supported did you feel by your social network (eg, friends and family)?” and “Currently, how supported do you feel by your social network?” Participants used a 7-point Likert scale from 1 (none) to 7 (very much supported) to report the extent to which they felt socially supported at each time frame. Each participant’s score for perceived social support prior to the pandemic was subtracted from their score for current perceived social support to create the variable “change in perceived social support,” such that higher values indicate increased perceived support from pre–COVID-19 to during COVID-19. Notably, our “change in perceived social support” variable was significantly negatively associated with loneliness (*r*=–0.22, *P*=.003), lending support to the notion that this change score adequately captured the extent to which participants felt their social support had changed during COVID-19.

##### Pandemic-Related Stress

We operationalized pandemic-related emotional well-being as pandemic-related stress, which was assessed with two questions: “In general, what is the level of distress you have experienced with COVID-19 related to social disruptions?” and “What is your overall level of stress related to the COVID-19 outbreak?” These questions were scored on a 7-point Likert scale from 1 (no distress or no impact) to 7 (extreme distress or extreme impact). These two questions were significantly positively associated in our sample (*r*=0.71, *P*<.001), lending support to the validity of this construct of pandemic-related stress. The internal consistency for items on this scale was good (*α*=.83).

#### Psychological Distress Measures

##### Loneliness

To assess loneliness, we administered the UCLA (University of California, Los Angeles) Loneliness Questionnaire [35], which is a 20-item self-report scale. Participants were asked to indicate how often each of the statements is descriptive of them (eg, “I feel isolated from others”). For the purposes of this study, one item—“I find myself waiting for people to call or write”—was modified to reflect more current communication practices: “I find myself waiting for people to call, text, message or otherwise contact me.” Responses were recorded on a scale from 0 (I never feel this way) to 3 (I often feel this way) and were summed. This scale was validated with a college student sample [35], and the internal consistency for items in the scale was excellent in this study’s sample (*α*=.95).

##### General Distress

To measure general emotional distress not necessarily attributable to the COVID-19 pandemic, we administered the Anhedonic Depression scale from the Mood and Anxiety Symptom Questionnaire (MASQ-AD). The MASQ-AD is a 22-item self-report scale that measures depressive symptomology [36]. Participants are presented with 22 items representing feelings, sensations, problems, and experiences (eg, “Felt like nothing was very enjoyable”) and are asked to report the extent to which they have experienced each item in the past week, using a 5-point Likert scale from 1 (not at all) to 5 (extremely). Items are summed to compute a total depression score. Due to the study taking place online and resulting ethical considerations, we omitted the suicidal ideation item, bringing the total number of items to 21 and the highest possible score to 105 instead of 110. Of note, this scale has been validated with three student samples and an adult sample [36]. The internal consistency for items on this scale in this sample was good (*α*=.83).

##### Social Anxiety

To measure a form of social distress not necessarily attributable to the COVID-19 pandemic, we administered the Social Interaction Anxiety Scale (SIAS) [37]. The SIAS is a 21-item self-report scale that measures the degree to which individuals experience anxiety specific to social interactions (eg, “I have difficulty talking with other people”). Participants indicated “the degree to which you feel the statement is characteristic or true of you” on a 5-point Likert scale from 0 (not at all characteristic or true of me) to 4 (extremely characteristic or true of me). Scores were summed, with possible scores ranging from 0 to 84. This scale was validated with college student, community, and clinical samples [37]. The internal consistency for items in this scale in this sample was good (*α*=.86).

## Results

### Sample

A total of 183 participants were recruited from undergraduate psychology courses at a private university in the Midwestern United States to participate in a study on emotions and social media. The final sample of 176 excluded 16 individuals who did not complete any of the measures in this study. Participant ages ranged from 18 to 23 years (mean 20.00, SD 1.26). Out of 176 participants, 54.0% (n=95) identified as women and 4.5% (n=8) identified as Hispanic or Latinx. With regard to race, our participants identified as follows: 44.9% (n=79) White, 26.7% (n=47) Asian, 19.9% (n=35) Black, and 8.5% (n=15) multi-racial.

### Analytic Overview

First, we provided descriptive statistics for each component of SNS use (ie, weekly frequency, time per day, habit, engagement, enjoyment, addiction, average emotional impact, and COVID-19–related SNS content) both before and during COVID-19, as well as pandemic-related social well-being (ie, changes in perceived social support), pandemic-related emotional well-being (ie, pandemic-related stress), and the three forms of psychological distress (ie, loneliness, depression, and social anxiety). We also presented Pearson zero-order correlations between the components of SNS use at both time frames (ie, pre– and during COVID-19). Then to assess effects of gender and race, we conducted a factorial multivariate analysis of variance, such that the components of SNS use were predicted by race and gender across the two time frames.

Aim 1 was to examine how SNS use has changed from pre–COVID-19 to during COVID-19, by comparing the means of the seven components of SNS use from pre–COVID-19 to during COVID-19 via a series of paired-sample *t* tests. We did not examine COVID-19–related SNS content because this construct was only assessed once during COVID-19.

Aim 2 was to examine how changes in components of SNS use during the pandemic were related to pandemic-related social well-being (ie, change in perceived social support) and pandemic-related emotional well-being (ie, pandemic-related stress). We created a residualized variable for each component of SNS use for which we assessed change, with each of the resulting variables representing the component of SNS use during COVID-19 that cannot be explained or predicted by the same component of SNS use pre–COVID-19; we will call this “change in SNS use components.” We conducted Pearson correlations between the change in SNS use components as well as COVID-19 SNS content and pandemic-related social and emotional well-being. Then, we conducted two linear regressions where we simultaneously entered the change in SNS use components and general distress (ie, depression) to predict pandemic-related social and emotional well-being. This allowed us to examine which changes in SNS use were uniquely related to the two outcomes while controlling for general distress.

Finally, Aim 3 was to examine how loneliness was associated with components of SNS use during the pandemic specifically and with change in SNS use from pre–COVID-19 to during the COVID-19 pandemic. First, we conducted zero-order Pearson correlations between the eight SNS components during COVID-19 and loneliness. Then, to examine unique effects of the SNS use components during COVID-19 on loneliness, we entered the eight SNS components simultaneously to predict loneliness. We also included social anxiety as a covariate so that effects were specific to loneliness and were not better explained by social anxiety. Next, to assess how changes in SNS use during COVID-19 are related to loneliness, we conducted Pearson correlations between loneliness and the seven “change in SNS use components” as well as COVID-19 SNS content. Finally, to assess unique effects of changes in SNS use on loneliness, we conducted a linear regression in which we entered the changes in SNS use components and COVID-19 SNS content and social anxiety simultaneously to predict loneliness.

### Descriptive Statistics and Correlations

Descriptive statistics for the eight components of SNS use for both time frames are presented in Table 1. Overall, these values are similar to existing work utilizing student samples [11,23]. With regard to descriptive statistics for the psychological distress measures, loneliness was lower than would be expected in a university sample (mean 23.09, SD 13.63) [24,27]. Levels of depressive symptoms were similar to other student samples (mean 56.76, SD 11.02) [36], and the sample can be characterized as having low levels of depression [25,28], although there was a wide range of values, including some above established clinical cutoff values [38]. Social anxiety was higher than typical in a student sample, but lower than would be expected in clinical samples (mean 33.80, SD 12.52) [26,29,30], indicating somewhat moderate levels of social anxiety in our sample, on average.

Zero-order Pearson correlations between the SNS use components pre– and during COVID-19 are presented in Table 2. Pre–COVID-19, correlations between SNS components ranged from –0.07 to 0.51 (mean 0.22, SD 0.17). During COVID-19, correlations between SNS components ranged from –0.13 to 0.56 (mean 0.25, SD 0.19). Test-retest correlations between time frames for each of the seven SNS components were generally large, ranging from 0.66 to 0.88 (mean 0.77, SD 0.07). There were not significant effects of gender (*F*_1,163_=1.48, *P*=.12) or race (*F*_3,163_=0.94, *P*=.59) on any of the components of SNS use.

**Table 1:** Descriptive statistics for social networking site (SNS) use pre–COVID-19 and during COVID-19.

Components of SNS use		Mean (SD)
**Pre–COVID-19**		
	Weekly frequency^a^	24.12 (6.60)
	Time per day (minutes)^b^	115.83 (113.53)
	Habit^c^	3.95 (0.82)
	Enjoyment^c^	3.61 (0.65)
	Engagement^c^	3.44 (0.93)
	Addiction^c^	8.98 (5.40)
	Average emotional impact^d^	4.29 (0.38)
**During COVID-19**		
	Weekly frequency^a^	24.57 (7.41)
	Time per day (minutes)^b^	196.38 (162.33)
	Habit^c^	4.11 (0.87)
	Enjoyment^c^	3.53 (0.77)
	Engagement^c^	3.52 (1.04)
	Addiction^c^	10.55 (6.02)
	Average emotional impact^d^	4.18 (0.50)
	COVID-19 SNS content (%)^e^	42.57 (22.89)

^a^Items were scored on an experimenter-generated 8-point Likert scale from 1 (never) to 8 (7+ times per day). Summed scores could range from 7 to 56.

^b^Participants were directly asked to report “how much time in a typical day” in the given time frame they had used each of the seven SNSs.

^c^Items were scored on a 5-point Likert scale from 1 (strongly disagree) to 5 (strongly agree). Averaged scores could range from 1 to 5.

^d^For each activity, participants indicated whether they had engaged in it during the given time frame. For all activities endorsed, participants indicated what impact each had on their emotions, on average, during the given time frame on a 7-point Likert scale from 1 (made me feel really bad) to 7 (made me feel really good). Scores were summed and divided by the total number of activities the participant endorsed.

^e^Participants reported what percentage they felt their SNS content was pandemic related in a text box.

**Table 2:** Correlation analysis (Pearson zero-order r and two-tailed *P* value) among social networking site (SNS) components pre–COVID-19 and during COVID-19.

Variable			Weekly frequency		Time per day		Habit		Enjoyment		Engagement		Addiction		Average emotional impact
**Weekly frequency pre–COVID-19**															
	*r*	1		0.42^a^		0.43^a^		0.24^a^		0.29^a^		0.36^a^		0.01	
	*P* value	—^b^		<.001		<.001		.002		<.001		<.001		.90	
**Weekly frequency during COVID-19**															
	*r*	1		0.41^a^		0.34^a^		0.25^a^		0.30^a^		0.25^a^		–0.07	
	*P* value	—		<.001		<.001		<.001		<.001		<.001		.36	
**Weekly frequency between pre– and during COVID-19**															
	*r*	0.88^a^		—		—		—		—		—		—	
	*P* value	<.001		—		—		—		—		—		—	
**Time per day pre–COVID-19**															
	*r*	0.42^a^		1		0.26^a^		0.19^a^		0.09		0.30^a^		0.03	
	*P* value	<.001		—		<.001		.03		.35		<.001		.61	
**Time per day during COVID-19**															
	*r*	0.41^a^		1		0.25^a^		0.17^a^		0.22^a^		0.19^a^		–0.04	
	*P* value	<.001		—		.002		.03		.006		.03		.88	
**Time per day between pre– and during COVID-19**															
	*r*	—		0.84^a^		—		—		—		—		—	
	*P* value	—		<.001		—		—		—		—		—	
**Habit pre–COVID-19**															
	*r*	0.43^a^		0.26^a^		1		0.42^a^		0.56^a^		0.33^a^		0.22^a^	
	*P* value	<.001		<.001		—		<.001		<.001		<.001		.004	
**Habit during COVID-19**															
	*r*	0.34^a^		0.25^a^		1		0.46^a^		0.51^a^		0.32^a^		0.06	
	*P* value	<.001		.002		—		<.001		<.001		<.001		.56	
**Habit between pre– and during COVID-19**															
	*r*	—		—		0.75^a^		—		—		—		—	
	*P* value	—		—		<.001		—		—		—		—	
**Enjoyment pre–COVID-19**															
	*r*	0.24^a^		0.19^a^		0.42^a^		1		0.51^a^		0.19^a^		0.29^a^	
	*P* value	.002		.03		<.001		—		<.001		.01		<.001	
**Enjoyment during COVID-19**															
	*r*	0.25^a^		0.17^a^		0.46^a^		1		0.52^a^		0.09		0.35^a^	
	*P* value	<.001		.03		<.001		—		<.001		.26		<.001	
**Enjoyment between pre– and during COVID-19**															
	*r*	—		—		—		0.78^a^		—		—		—	
	*P* value	—		—		—		<.001		—		—		—	
**Engagement pre–COVID-19**															
	*r*	0.29^a^		0.09		0.56^a^		0.51^a^		1		0.13		0.21^a^	
	*P* value	<.001		.35		<.001		<.001		—		.09		.006	
**Engagement during COVID-19**															
	*r*	0.30^a^		0.22^a^		0.51^a^		0.52^a^		1		0.23^a^		0.13	
	*P* value	<.001		.006		<.001		<.001		—		.003		.06	
**Engagement between pre– and during COVID-19**															
	*r*	—		—		—		—		.79^a^		—		—	
	*P* value	—		—		—		—		<.001		—		—	
**Addiction pre–COVID-19**															
	*r*	0.36^a^		0.30^a^		0.33^a^		0.19^a^		0.13		1		–0.13	
	*P* value	<.001		<.001		<.001		.01		.09		—		.09	
**Addiction during COVID-19**															
	*r*	0.25^a^		0.19^a^		0.32^a^		0.09		0.23^a^		1		—	
	*P* value	<.001		.03		<.001		.26		.003		—		—	
**Addiction between pre– and during COVID-19**															
	*r*	—		—		—		—		—		0.72^a^		—	
	*P* value	—		—		—		—		—		<.001		—	
**Average e** **motional** **impact pre–COVID-19**															
	*r*	0.01		0.03		0.22^a^		0.29^a^		0.21^a^		–0.13		1	
	*P* value	.90		.61		.004		<.001		.006		.09		—	
**Average e** **motional** **impact during COVID-19**															
	*r*	–0.07		–0.04		0.06		0.35^a^		0.13		–0.26^a^		1	
	*P* value	.36		.88		.56		<.001		.06		<.001		—	
**Average e** **motional** **impact between pre– and during COVID-19**															
	*r*	—		—		—		—		—		—		0.66^a^	
	*P* value	—		—		—		—		—		—		<.001	

^a^The correlation is significant at a significance level of .05 (two-tailed).

^b^Not applicable.

### Aim 1. How Components of SNS Use Changed From Before to During COVID-19

Consistent with our hypothesis, there was an increase in daily time spent on SNSs (*t_169_*=5.53, *d*=0.42, *P*<.001), habitual use of SNSs (*t_173_*=3.60, *d*=0.27, *P*<.001), and SNS addiction (*t_173_*=4.96, *d*=0.38, *P*<.001) during COVID-19 compared to pre–COVID-19. In addition, the average impact of endorsed SNS activities on emotions became more negative (*t_172_*=–3.76, *d*=–0.29, *P*<.001). Inconsistent with our hypotheses, enjoyment on SNSs decreased (*t_172_*=–2.10, *d*=–0.16, *P*=.04). Weekly frequency of SNS use (*t_174_*=1.74, *d*=0.14, *P*=.08) and engagement with SNSs (*t_173_*=1.53, *d*=0.12, *P*=.13) did not significantly change during COVID-19.

### Aim 2. How Changes in Components of SNS Use Are Related to Pandemic-Related Social and Emotional Well-being

#### Change in Perceived Social Support

First, consistent with our hypothesis, we found that change in weekly frequency, change in time per day, and change in addiction were each positively associated with increased social support. Contrary to our hypothesis, change in engagement was also positively associated with increased social support (Table 3). That is, compared to pre–COVID-19, those who visited SNSs more frequently during COVID-19*,* those who spent more time on SNSs during COVID-19, those who experienced more SNS addiction during COVID-19, and those who were more engaged on SNSs during COVID-19 endorsed greater increases in perceived social support during COVID-19. Inconsistent with our hypothesis, changes in SNS habit, average emotional impact, and COVID-19 SNS content were not significantly associated with perceived change in social support during the pandemic. When examining all seven “change in SNS use components,” COVID-19 SNS content, and depression in the same model predicting change in perceived social support, no associations were significant. These findings suggest that no changes in SNS use from pre– to during COVID-19 uniquely predict change in perceived social support.

**Table 3:** Pearson zero-order correlations between changes in social networking site (SNS) use components and pandemic-related social and emotional well-being measures.

Variable^a^			*r*	*P* value	
**Change in perceived social support**					
	Change in weekly frequency	0.24^b^			.007
	Change in time per day (minutes)	0.20^b^			.02
	Change in habit	0.15			.08
	Change in enjoyment	0.10			.30
	Change in engagement	0.20^b^			.02
	Change in addiction	0.18^b^			.02
	Change in average emotional impact	0.04			.46
	COVID-19 SNS content	–0.01			.68
**Pandemic-related stress**					
	Change in weekly frequency	0.06			.33
	Change in time per day (minutes)	0.07			.38
	Change in habit	0.05			.48
	Change in enjoyment	–0.02			.79
	Change in engagement	0.12			.13
	Change in addiction	0.23^b^			.002
	Change in average emotional impact	0.07			.39
	COVID-19 SNS content	0.20^b^			.006

^a^These analyses utilizing change components used residualized SNS use variables.

^b^The correlation is significant at a significance level of .05 (two-tailed).

#### Pandemic-Related Stress

Consistent with our hypothesis, we found that pandemic-related stress was significantly positively associated with change in addiction and COVID-19 SNS content. Inconsistent with our hypothesis, pandemic-related stress was not associated with changes in frequency, time, habit, engagement, or average emotional impact of SNS use (Table 3). When predicting pandemic-related stress from the seven “change in SNS use components,” COVID-19 SNS content, and general distress (covariate), these results showed the same pattern of findings (addiction: *β*=0.07, *t_159_*=2.90, *P*=.004; COVID-19 SNS content: *β*=0.01, *t_159_*=2.44, *P*=.02). These findings suggest that both increased addictive SNS use and the percentage of one’s SNS content related to the pandemic are associated with pandemic-related stress, even after taking into account the other SNS use components and general distress.

#### Loneliness

We assessed how loneliness is associated with SNS use during the pandemic. Consistent with our hypothesis, we found that loneliness was positively associated with addiction during COVID-19 and negatively associated with engagement and the average emotional impact of SNSs during COVID-19. Inconsistent with our hypotheses, loneliness was not significantly associated with weekly frequency, time per day, habit, enjoyment, or COVID-19 SNS content (Table 4). However, when examining all eight SNS components during COVID-19 and social anxiety (as a covariate) simultaneously, only addiction was significant (*β*=0.50, *t_160_*=2.78, *P*=.006). These results suggest that it is only addictive SNSs that are uniquely associated with loneliness during COVID-19.

Second, we assessed how loneliness is associated with changes in SNS use during the pandemic. Consistent with our hypothesis, we found that loneliness was negatively associated with change in engagement. That is, those who were lonelier endorsed less SNS engagement during COVID-19 compared to their endorsed engagement pre–COVID-19. Contrary to our hypothesis, loneliness was also negatively associated with change in habit. Also inconsistent with hypotheses, loneliness was not significantly associated with changes in weekly frequency, time per day, enjoyment, addiction, average emotional impact, or COVID-19 SNS content (Table 4). When we considered the seven “change in SNS use components” simultaneously while controlling for social anxiety, change in habit was significant (*β*=–4.86, *t_158_*=–2.67, *P*=.001), engagement was not significant (*β*=–2.11, *t_158_*=–1.29, *P*=.20), and addiction was significant (*β*=0.49, *t_158_*=1.98, *P*=.049). In this way, addiction was revealed in the linear regression model as a previously suppressed variable that required a more powerful test with decreased standard error to be revealed. These results suggest that loneliness was uniquely associated with using SNSs less habitually and being more addicted to SNSs during the pandemic.

**Table 4:** Pearson zero-order correlations between loneliness and social networking site (SNS) use components during COVID-19 and changes in SNS use components from pre– to during COVID-19.

Variable		*r*	*P* value
**SNS use during** **COVID-19^a^**			
	Change in weekly frequency	0.07	.34
	Change in time per day (minutes)	0.10	.19
	Change in habit	–0.07	.17
	Change in enjoyment	–0.10	.18
	Change in engagement	–0.16^b^	.04
	Change in addiction	0.26^b^	<.001
	Change in average emotional impact	–0.19^b^	.01
	COVID-19 SNS content	0.07	.34
**Changes in SNS use from pre– to during COVID-19^c^**			
	Change in weekly frequency	0.07	.39
	Change in time per day (minutes)	0.00	.99
	Change in habit	–0.21^b^	.01
	Change in enjoyment	–0.08	.27
	Change in engagement	–0.15^b^	.04
	Change in addiction	0.12	.31
	Change in average emotional impact	0.02	.98

^a^These analyses used variables assessed with the time frame of during COVID-19.

^b^The correlation is significant at a significance level of .05 (two-tailed).

^c^These analyses used residualized SNS use variables.

## Discussion

This investigation examined how the COVID-19 pandemic was associated with changes in SNS use and how these changes were associated with psychological outcomes in a college student sample. This study expands upon the literature in several important ways. First, rather than assessing only frequency of SNS use, we examined how multiple components of SNS use (ie, weekly frequency, time per day, habit, engagement, enjoyment, addiction, average emotional impact, and COVID-19–related SNS content) changed from pre– to during COVID-19, and how these changes in SNS use were related to social and emotional well-being. Second, this investigation assessed how these components of SNS use were related to loneliness during a global pandemic when rates of loneliness are believed to be elevated. Lastly, to our knowledge, this was the first investigation to examine the perceived impact of engagement in SNS activities on people’s emotions and how this was associated with the COVID-19 pandemic.

The study’s first aim was to examine how SNS use changed from pre– to during COVID-19. Mostly consistent with hypotheses based on Turel and Serenko’s [12] path model, changes in SNS use, including time spent on SNSs; habitual SNS use; and SNS addiction increased, while enjoyment on SNSs decreased and the average emotional impact of SNS activities became more negative. These findings suggest that individuals’ increased thoughts and behaviors toward SNSs during COVID-19 (ie, spending more time on SNSs, using them more habitually, and experiencing greater addiction) could be maladaptive and are associated with more negative emotional experiences.

The study’s second aim was to investigate how changes in components of SNS use during the pandemic were related to pandemic-related social and emotional well-being. Contrary to our hypotheses that increased SNS use would be negatively associated with pandemic-related social and emotional well-being, greater increases in perceived social support during COVID-19 were associated with (1) more frequent SNS use, (2) more time spent on SNSs, (3) greater SNS addiction, and (4) greater engagement with SNSs during COVID-19. However, when all SNS components were taken into consideration, none were significantly associated with perceived social support. This suggests that no one way of using SNSs (ie, using them more frequently, more addictively, etc) uniquely accounted for increased perceptions of social support during COVID-19. Consequently, results should be interpreted with caution. Nonetheless, these findings provide some evidence that SNS use during the pandemic could be socially adaptive and might create a space for individuals to feel more socially connected. SNS addiction during COVID-19 and the extent to which one’s SNS content was related to the pandemic were associated with greater pandemic-related stress, controlling for general distress, consistent with hypotheses.

Overall, the associations between components of SNS use and pandemic-related well-being were mixed. Greater perceived social support during COVID-19 was associated with using SNSs more frequently and for more time, as well as reporting greater SNS engagement and addiction. In contrast, SNS addiction during COVID-19 and exposure to COVID-19–related SNS content were each associated with decreases in pandemic-related social and emotional well-being. And compared to pre–COVID-19, individuals during COVID-19 reported enjoying SNSs less and experiencing greater negative impacts of SNS activities on their emotions. Overall, these results suggest that, despite individuals using SNSs more frequently and for more time during the pandemic, use of SNSs during COVID-19 was associated with mixed social outcomes and largely negative emotional outcomes.

The study’s third aim was to examine how loneliness was associated with SNS use during the pandemic. Consistent with hypotheses, higher levels of loneliness were significantly associated with SNS activities during COVID-19 having a negative emotional impact. Loneliness was positively associated with SNS addiction during COVID-19 and negatively associated with engagement in SNSs during COVID-19. Importantly, addictive SNS use during COVID-19 was significantly related to loneliness even after accounting for the other SNS use components and social anxiety. Inconsistent with hypotheses, loneliness was associated with reductions in habitual SNS use and engagement on SNSs from pre– to during COVID-19. However, when simultaneously considering how all SNS components and social anxiety were associated with loneliness, only reductions in habit remained significant, and addictive SNS use became significant.

Although these findings illustrate associations between loneliness and various components of SNS use, further research is needed to determine directionality between these constructs. On the one hand, it is possible that people were lonely during the pandemic because they were not using SNSs as habitually as they once had. On the other hand, it could be that those who were lonely during COVID-19 were aware of the negative impact of SNS use on their emotions and mental health and, therefore, chose to engage with SNSs less habitually during the pandemic. In a sense, a decrease in SNS habit during COVID-19 could serve as a protective mechanism for those high in loneliness. Although findings showed that decreases in habit were associated with loneliness, increases in SNS addiction during the pandemic were also associated with loneliness. Perhaps individuals who were lonely during COVID-19 stopped using SNSs habitually and used them more addictively instead, an outcome that may occur if individuals wish to use SNSs less but still find themselves turning to them.

Our additional findings that loneliness was associated with SNS activities having a more negative, or less positive, emotional impact may shed important insight into the role of SNSs on loneliness. Research suggests that loneliness causes increased SNS use, and not that SNS use causes increased loneliness [21]. Despite lonely people using SNSs more than others, increased loneliness is also correlated with experiencing greater negative influences of SNSs on emotions. Again, further research is needed to understand the temporal relations between these constructs. It could be that when individuals who are lonely turn to SNSs to receive social stimuli, (1) this exposure leads to social comparison experiences that result in negative emotions (ie, seeing pictures of peers spending time together) or (2) they may not reap the same emotional benefits from them as do those who are less lonely. Future research should examine these possibilities to begin to elucidate how emotions for those who are lonely are implicated in SNS use.

Interestingly, across analyses examining our three aims, addictive SNS use was the SNS activity that was most consistently significant in our models, highlighting its potential importance in predicting well-being. Namely, addiction significantly increased from pre– to during COVID-19. Addiction was also associated with increases in perceived change in social support during COVID-19, greater pandemic-related stress, and greater loneliness. These findings suggest that SNS users should be aware of their addictive SNS tendencies and be cognizant of how this addictive use may be associated with their well-being (eg, noting that addictive SNS use makes them feel more socially connected, but also more stressed). Future research should continue to explore the role of SNS addiction in individuals’ everyday lives and emotional experiences, especially during times of global health crises when SNS use seems to increase.

This investigation highlights potential clinical implications. During COVID-19, therapy clients, like this study’s sample, may report that their use of SNSs during COVID-19 has increased. In these cases, it may be helpful for mental health providers to note that increased SNS use during COVID-19 has been linked to mixed outcomes, at least in a college student sample. Mental health providers could help their clients examine when SNS use may be adaptive versus maladaptive and when clients, for example, should pursue other social outlets (eg, having in-person conversations). This is consistent with cognitive behavioral therapies, which place emphasis on helping clients engage in behaviors that have an emotion-boosting effect and limiting behaviors that negatively influence emotions [39]. Examining SNS use in therapy seems particularly critical for clients who report elevated loneliness, since they may be using SNSs more than others.

Furthermore, these findings suggest utility in assessing and monitoring for SNS addiction specifically. Although SNS addiction, as assessed in this study, was associated with increased perceived social support, it was also associated with greater stress and loneliness. An important avenue for future research is to examine at which levels SNS addiction causes clinically significant distress or impairment, which are requirements for receiving formal diagnoses of addictive disorders [40]. It will also be important to establish which treatments are best suited for treating SNS addiction since there are not currently any empirically supported treatments for it. Of note, experts have posited that treatment should center on controlling SNS use, rather than abstaining from it, since SNSs have become an integral and unavoidable part of life [39,40], which may be especially true for college-aged individuals. In addition, it might be useful to provide psychoeducation to clients about SNS addiction.

This investigation has several limitations. Most notably, since data were collected about one month into the pandemic, it is not known whether these trends of SNS use have continued throughout the pandemic. However, given the initial surge in use in this sample, we would expect trends in SNS use to persist on the premise that—as can be seen in this investigation as well as others—SNS use is habitual [12]. Both SNS addiction and using SNSs as a means to connect with others are associated with habitual SNS use [12,41]. Therefore, given the increase in SNS addiction seen in this sample and our theorizing that SNSs were used as tools for social connection, it is not surprising that our data showed an increase in habit near the start of the pandemic. Once habits are formed, they are very difficult to break [42], leading us to think that these trends in SNS use witnessed at the beginning of the pandemic would remain today.

It is also important for SNS research to utilize designs other than retrospective reports, which can be biased and more difficult for participants to accurately complete [43]. For example, prospective longitudinal research could be utilized to examine how SNS use and its associations with well-being evolve over the course of disease outbreaks. Additionally, because this investigation focused on college students, the findings may not generalize to peers who do not attend college or to older samples. Consequently, this study did not shed light on how SNS use during disease outbreaks is related to loneliness and various indices of well-being across the adult lifespan. We expect that use of SNSs during the pandemic would be related to positive outcomes, particularly in older adults. Older adults have been shown to have generally positive feelings toward SNSs, and SNS use in this population is associated with greater well-being and less loneliness [43,44].

In a world where an increasing amount of time and social interactions are occurring in an online sphere, it is imperative to investigate and understand the role of SNSs on social and emotional well-being during times of crisis. Findings from this investigation highlight both benefits and disadvantages to SNS use, underscoring the nuanced and multifaceted nature of the correlates of these sites with well-being. Although the COVID-19 pandemic may be one of the first globally salient incidents that has erupted since the widespread adoption of SNS use, it is unlikely to be the last. It is hoped that findings from this investigation will advise SNS users on how to best cope with the COVID-19 pandemic and any future pandemics as well. This study and those like it are only beginning to help us truly understand how SNS use is associated with our everyday social and emotional well-being during stressful and trying times.

## Abbreviations

MASQ-AD: Anhedonic Depression scale from the Mood and Anxiety Symptom Questionnaire
SIAS: Social Interaction Anxiety Scale
SNS: social networking site
UCLA: University of California, Los Angeles
